# Factor structure and psychometric properties of the Chinese version of the Intuitive Exercise Scale for young adults

**DOI:** 10.3389/fpsyg.2025.1546015

**Published:** 2025-02-19

**Authors:** Dongzhe Shi, Fangyan Lv, Jingbin Tan, Dingguo Gao

**Affiliations:** ^1^Department of Sports Science and Physical Education, Guangzhou Xinhua University, Guangzhou, China; ^2^School of Marxism, Sun Yat-Sen University, Guangzhou, China; ^3^Department of Psychology, Sun Yat-Sen University, Guangzhou, China

**Keywords:** intuitive exercise, physical activity, psychometrics, young adults, Chinese translation

## Abstract

**Background:**

The Intuitive Exercise Scale (IEXS) is a scale designed to evaluate the positive relationship with exercise, including their ability to tune in to bodily cues, maintain mindfulness while moving, and utilize diverse movement patterns. As that the psychometric properties of the IEXS have not been thoroughly established across cultures and various age groups, the purpose of this study is to evaluate the applicability of the translated IEXS among young Chinese adults.

**Methods:**

We translated and culturally adapted the IEXS for a sample of 630 Chinese young adults (*M* = 19.61 *SD* = 1.13) who agreed to complete the scale online. The psychometric evaluation included item analysis, exploratory and confirmatory factor analysis (EFA and CFA), and assessments of reliability, predictive validity, and criterion validity.

**Results:**

The mean IEXS score was 47.23 (SD = 7.28), and EFA identified four latent constructs: emotional exercise, exercise rigidity, body trust, and mindful exercise. The CFA confirmed the model’s fit (CFI = 0.98; SRMR = 0.06). Item-total correlations ranged from 0.639 to 0.753, with item loadings between 0.635 and 0.760. Also, the reliability metrics demonstrated strong internal consistency (Cronbach’s *α* = 0.869) and test–retest stability (ICC = 0.721, *p <* 0.01). Predictive validity was confirmed through significant correlations with the Physical Activity Rating Scale (PARS-3), and criterion validity showed moderate correlations (0.342–0.615) with validated scales.

**Conclusion:**

The Chinese IEXS is a robust and valid tool for assessing intuitive exercise tendencies in Chinese young adults.

## 1 Introduction

Engaging in physical activity provides a host of physical and mental health advantages (Chekroud et al., 2018; Herbert et al., 2020), including improved strength, endurance, and coordination, and also contributes to preventing chronic diseases including diabetes, heart disease, and cancer (Kraak and Story, 2010; Murtagh and Ludwig, 2011). Furthermore, regular physical activity also alleviates psychological challenges such as depression, anxiety, and stress (Donaghy, 2007; Hu et al., 2020; Wilhite et al., 2023; Kandola et al., 2020). However, studies worldwide consistently reveal that many adults fail to meet recommended physical activity levels (Grasdalsmoen et al., 2020; Rocliffe et al., 2024; Schlack et al., 2021), with college students being particularly susceptible to inactivity (Malagodi et al., 2024; Teuber et al., 2024). The popular phrase “Crispy College Students” highlights the vulnerability of this demographic to health issues, suggesting long-standing challenges in their exercise-related behaviors and attitudes (Zhou et al., 2024). This trend highlights the significance of achieving a deeper comprehension and tacking the root causes affecting physical activity within this group.

Simultaneously, dysfunctional eating and exercise behaviors (Gorrell et al., 2021; Reel et al., 2016a; Reel et al., 2016b), have become more prevalent, posing risks for obesity (Carraça et al., 2021; Hayes et al., 2018), eating disorders (Godoy-Izquierdo et al., 2023; Monthuy-Blanc et al., 2023), and other mental health concerns (Coniglio et al., 2022; Cuesta-Zamora et al., 2021). Besides, body image plays a central role in shaping dietary and exercise habits, often influencing the likelihood of developing eating disorders (Berengüí et al., 2024; Brown and Tiggemann, 2022; Dane and Bhatia, 2023) and obesity (Robertson et al., 2021; Stabouli et al., 2021). Specifically, negative body image perceptions frequently drive individuals toward extreme dietary restrictions or binge eating, heightening the risk of eating disorders (Shriver et al., 2020). Congruently, dysfunctional exercise patterns, including excessive exercise or insufficient exercise, further contribute to obesity and related chronic conditions (Monthuy-Blanc et al., 2023). These unhealthy behaviors often stem from dissatisfaction with body weight or shape, leading to impractical or harmful approaches to diet and physical activity (Duarte et al., 2021). Understanding these interconnected issues is essential for promoting healthier behaviors among vulnerable populations. Young adults, being in a critical developmental stage, are more influenced by social and peer pressures, increasing their risk of unhealthy habits. They also often lack adequate health knowledge and self-management skills, making them a significant part of the vulnerable population.

Intuitive exercise emphasizes responding to bodily signals, such as pain, soreness, or fatigue, to guide physical activity rather than adhering to rigid routines that may lead to injury or illness (Reel and Miyairi, 2012). This approach fosters awareness of the body’s movements and sensations, using these as cues to start or stop exercising (Reel, 2015). By cultivating a mindful and positive relationship with physical activity, intuitive exercise addresses issues like inactivity, excessive exercise, addiction, or dysfunctional behaviors (Reel et al., 2016a; Reel et al., 2016b). It closely links to body self-image through factors like interoceptive awareness, emotional exercise, body trust, and mindful movement, all of which shape people’s cognitive and emotional experiences of their bodies. For example, the intuitive movement encourages individuals to make decisions about exercise based on bodily cues rather than external pressures, promoting mindfulness and a healthier body image. Research suggests a negative correlation between interoceptive awareness and body dissatisfaction, indicating that heightened awareness can enhance body image. This mindful approach to exercise helps individuals develop a balanced, self-aware perspective on physical activity (Reel et al., 2016a; Reel et al., 2016b).

Reel et al. (2016a) and Reel et al. (2016b) first created the Intuitive Exercise Scale (IEXS) to measure intuitive exercise, exhibiting strong internal consistency reliability as assessed by Cronbach’s alpha (0.86–0.91) in adult community samples. Intuitive exercise offers potential benefits, particularly in treating and recovering from eating disorders (Reel, 2015). By attuning to their body’s internal signals, individuals can better meet their physical needs and avoid excessive exercise or related injuries. During recovery, intuitive exercise serves as a flexible tool to help patients rebuild a healthy relationship with food and physical activity (Voelker et al., 2021). Although research on the scale’s application remains limited, studies have shown its usefulness. For instance, Yon et al. (2022) indicated that the COVID-19 pandemic negatively impacted young adults’ intuitive exercise levels and attitudes, highlighting the scale’s relevance in understanding exercise behaviors (Yon et al., 2022). Recent studies, such as one conducted in Italy, have further validated the IEXS, confirming its four-factor structure, internal consistency, and convergent validity (Mancin et al., 2024). Italian speakers engaged in physical activities reported higher levels of intuitive exercise, emphasizing the importance of cultural considerations in exercise and eating disorder research (Mancin et al., 2024). Expanding the scale’s application across diverse contexts can provide valuable insights into intuitive exercise and its benefits.

Applying the IEXS across cultural contexts may uncover differences influenced by cultural values, social norms, health perceptions, language nuances, and exercise habits. To ensure its effective use in diverse settings, researchers should conduct cross-cultural validation of the scale. This study therefore seeks to adapt the IEXS to the socio-economic and cultural context of China, providing a tailored version suitable for Chinese young adults. By assessing the scale’s applicability and feasibility within this demographic, the research aims to offer a reliable tool for evaluating physical activity and exercise behaviors in Chinese settings.

## 2 Materials and methods

### 2.1 Study design

Utilizing convenience sampling, a total of 654 college students from two universities in Guangzhou, Guangdong Province, China, were recruited for this study. The data was gathered via the use of an online survey platform: Wenjuanxing (in China). Questionnaires were considered invalid if the completion time was excessively brief (under 300 s) or if a participant provided uniform responses to all items (or followed a highly consistent response pattern) throughout, suggesting that the participant did not read the items or did not thoughtfully consider each one (Liu et al., 2023). In this sample, 13 participants identically provided their responses with completion times of less than 300 s. Meanwhile, 11 participants had incomplete data on Wenjuanxing, resulting in the exclusion of their data. Finally, sample 1 involved a total of 630 participants (*M*_age_ = 19.61, *SD* = 1.13) in final analyses, who were divided into two groups using an odd-even sorting method. This sample included 212 males (*M*_age_ = 19.74, *SD* = 1.35) and 418 females (*M*_age_ = 19.54, *SD* = 0.99). Data from the odd-numbered group (n = 315) were used for exploratory factor analysis (EFA), while the even-numbered group (*n* = 315) provided data for confirmatory factor analysis (CFA).

2 weeks after the initial survey, researchers randomly selected 220 participants as sample 2 from Sample 1 to reassess the IEXS for test–retest reliability. Every eligible participant was given a unique number, and then the selection of the required number of participants was carried out using a random number generator. The researchers selecting the participants were unaware of any identifying information about the potential participants until after the selection was completed. Using students’ IDs to match responses, they identified 218 valid surveys after removing duplicates submitted by participants who finished the survey multiple times. The final group included 94 males and 124 females, aged 18 to 21 years, with an average age of 19.28 ± 0.96 years.

The Human Subjects Protection Ethics Review Committee of the Psychology Department at Sun Yat-sen University approved the research (ethical number: 2025–0115-0375). Written informed consent was obtained from participants before they participated in the study. Additionally, participants were made aware of their right to leave the survey at any point by closing their browsers, ensuring their results would not be recorded.

### 2.2 Measures

#### 2.2.1 Chinese version of the intuitive exercise scale (IEXS-C)

The IEXS evaluates individuals’ relationships with exercise (Reel et al., 2016a; Reel et al., 2016b), focusing on aspects such as enjoyment, activity diversity, mindfulness, and responsiveness to bodily signals. The Chinese version of this scale makes up of 14 items divided into four dimensions: Emotional Exercise (5 items: 2, 3, 8, 9, 14) measures how exercise helps manage negative emotions; Body Trust (3 items: 4, 5, 7) assesses reliance on personal judgment regarding exercise type, intensity, and frequency; Exercise Rigidity (3 items: 10, 12, 13) evaluates adherence to varied exercise routines; and Mindful Exercise (3 items: 1, 6, 11) measures awareness of bodily signals to begin or stop exercising. A 5-point Likert scale (1 = Strongly Disagree, 5 = Strongly Agree) is employed to rate each item, with elevated scores corresponding to increased levels of intuitive exercise (Reel and Miyairi, 2012; Reel, 2015; Reel et al., 2016a; Reel et al., 2016b).

Notably, the IEXS was revised for use in Chinese through a structured process: (1) The research team obtained authorization from the original author; (2) A translation team of three psychologists (two males and one female) independently translated the IEXS items; (3) Items 8 and 10 presented challenges in translation, so the team sought clarification from the original author and subsequently developed the inaugural Chinese version of the IEXS, integrating their feedback into the process; (4) To ensure clarity and fluency, seven students (three males and four females, aged 18–22) were recruited to evaluate each item in the first Chinese version individually; (5) Based on their feedback, the translation team revised the first version, producing a second Chinese edition; (6) Two female English instructors performed a back-translation of the second rendition in Chinese into English. A comparison and iterative refinement were conducted among the original English text, the second Chinese adaptation, and the English retranslation, culminating in the creation of the final Chinese version of the IEXS (IEXS - C). Therefore, in accordance with the guidelines demonstrated in previous research (Ding et al., 2021), appropriate modifications were deliberated and consented upon by both translators and the authors to fit the cultural context, provided that functional and cultural equivalence was maintained.

#### 2.2.2 Additional measures

The IEXS is designed to assess individuals’ tendencies and effectiveness in practicing intuitive exercise, which is strongly related to physical activity and mental health. To assess its criterion validity, the study selected complementary scales that correlate to physical activity and mental health for preliminary analysis. These additional measures were chosen to explore the connections between intuitive exercise, overall physical well-being, and psychological health outcomes.

The Physical Activity Rating Scale (PARS-3), by Liang (1994), measures the intensity, duration, and frequency of physical activity. This scale uses a 5-point Likert scoring system for each component, with total scores ranging from 0 to 100, reflecting an individual’s level of physical activity. In this research, participants’ physical activity scores fell within the full range of the scale. The PARS-3 demonstrated strong reliability, with a Cronbach’s *α* coefficient of 0.82, confirming its consistency in assessing physical activity levels.

The Satisfaction with Life Scale (SWLS), which was first created by Diener et al. (1985) and then modified by Leung and Leung (1992), assesses the cognitive dimension of subjective well-being. Comprising five items, the scale uses a 7-point Likert format, ranging from “completely disagree” to “completely agree.” The scores for life satisfaction extend from 5 to 35, and greater scores signify increased satisfaction with life. In this study, participants’ scores extended across the whole range of the scale. The SWLS showed robust internal consistency, featuring a Cronbach’s *α* reliability coefficient of 0.85, validating its use for assessing life satisfaction in this context.

The Positive and Negative Affect Scale (PANAS), which was initially created by Watson et al. (1988) and subsequently updated by Huang et al. (2003), measures individuals’ emotional states through two subscales: Positive Affect (PA) and Negative Affect (NA). Each subscale contains 10 descriptive adjectives, resulting in a total of 20 items. Participants rate their emotions on a 5-point Likert scale, ranging from 1 (“nothing at all”) to 5 (“very strong”). The total score for each subscale ranges from 10 to 50, with higher scores reflecting stronger positive or negative emotional experiences. The scale showed robust internal consistency in the study with Cronbach’s *α* coefficients of 0.82 for PA, and 0.84 for NA.

#### 2.2.3 Social support

The Social Support Rating Scale (SSRS), developed by Xiao (1999), assesses an individual’s perceived social support. Furthermore, the scale makes up of 10 items, with subjective support measured by 4 items that capture emotional experiences and satisfaction with feeling respected, supported, and understood. Moreover, objective support includes 3 items assessing the tangible support received, while support utilization comprises 3 items evaluating how individuals utilize social support. The SSRS has proven to be both reliable and valid in studies involving Chinese young adults. In this research, the scale’s Cronbach’s α coefficient was 0.61, indicating acceptable reliability for measuring social support.

### 2.3 Data analysis

This study used SPSS version 26.0 to conduct item analysis, reliability analysis, and EFA, while AMOS version 21.0 was employed for CFA, equivalence testing, and latent mean analysis. The significance level was set at *p* < 0.05.

## 3 Results

### 3.1 Item analysis

Item analysis was performed by exploring the association between each item score and the total scale score. In the item analysis, the following metrics were calculated: mean with standard deviation, median with interquartile range, corrected item-total correlation, and squared multiple correlations (SMC). Based on their total scale scores, participants were ranked from highest to lowest, with the top and bottom 27% being divided into high and low groups for independent samples T-tests. The findings suggested that the item scores of the high-scoring group were considerably higher compared to the low-scoring group (*p* < 0.001). Additionally, Pearson correlation coefficients between the item scores and the average scores of their respective dimensions ranged from 0.50 to 0.74, all of which were statistically significant (*p* < 0.001). This indicated that all items were well differentiated and kept in the final version (see Table 1).

**Table 1:** Descriptive metrics and correlation indices.

Item	M ± SD	Median	Percentiles (25)	Percentiles (50)	Percentiles (75)	Squared multiple correlation (SMC)	Corrected item-total correlation	*t*
Item_1	3.76 ± 0.81	4	3	4	4	0.56	0.50	11.08
Item_2.	2.97 ± 0.85	3	2	3	4	0.59	0.65	17.00
Item_3	3.00 ± 0.88	3	2	3	4	0.62	0.67	18.05
Item_4	3.35 ± 0.87	3	3	3	4	0.58	0.68	17.63
Item_5	3.45 ± 0.83	4	3	4	4	0.62	0.70	17.92
Item_6	3.66 ± 0.79	4	3	4	4	0.70	0.51	10.32
Item_7	3.58 ± 0.79	4	3	4	4	0.50	0.66	15.91
Item_8	3.49 ± 0.88	4	3	4	4	0.54	0.71	19.18
Item_9	3.11 ± 0.85	3	3	3	3	0.51	0.66	17.44
Item_10	3.16 ± 0.81	3	3	3	4	0.54	0.69	16.28
Item_11	3.65 ± 0.75	4	3	4	4	0.70	0.55	11.48
Item_12	3.42 ± 0.80	3	3	3	4	0.56	0.70	16.59
Item_13	3.18 ± 0.84	3	3	3	4	0.50	0.65	16.51
Item_14	3.46 ± 0.81	4	3	4	4	0.57	0.74	20.00

### 3.2 Structure validity analysis

#### 3.2.1 Exploratory factor analysis

The first round of EFA was conducted on the 14 items from the sample’s odd group (*n* = 315). The results indicated a KMO value of 0.869 and Bartlett’s test of sphericity yielded a *χ*^2^ value of 2508.150 with 91 degrees of freedom, showing statistical significance at *p* < 0.001, which confirmed the appropriateness of the data for conducting factor analysis. Factors were extracted using principal component analysis according to the criterion of eigenvalues greater than 1, and the scree plot supported the extraction of four factors. However, item 14 did not align with the other items in its factor, so it was excluded from further analysis.

The remaining 13 items underwent a second round of EFA. The results revealed a KMO value of 0.851, Bartlett’s test of sphericity showing a *χ*^2^ value of 2266.972 and 78 degrees of freedom (*p* < 0.001), further supporting the data’s appropriateness for conducting factor analysis. Principal component analysis indicated that four factors were most appropriate for extraction, with eigenvalues of 5.226, 2.380, 1.197, and 1.092, accounting for a cumulative variance of 76.115%. Item loadings for the four factors were as follows: Emotional Exercise ranged from 0.71 to 0.83, Body Trust from 0.78 to 0.85, Exercise Rigidity from 0.74 to 0.84, and Mindful Exercise from 0.86 to 0.91. The final factor structure included the following items: Emotional Exercise (items 2, 3, 8, 9), Body Trust (items 4, 5, 7), Exercise Rigidity (items 10, 12, 13), and Mindful Exercise (items 1, 6, 11) (see Table 2).

**Table 2:** Factor loadings for four-factor solution of the IEXS (*n* = 315).

Item	1	2	3	4
Item 2	**0.83**	0.12	0.16	−0.04
Item 3	**0.83**	0.20	0.23	−0.03
Item 8	**0.71**	0.26	0.22	0.11
Item 9	**0.79**	0.17	0.16	0.09
Item 4	0.22	**0.85**	0.18	0.08
Item 5	0.26	**0.83**	0.24	0.11
Item 7	0.19	**0.78**	0.10	0.27
Item 10	0.31	0.25	**0.74**	0.02
Item 12	0.22	0.24	**0.78**	0.22
Item 13	0.18	0.06	**0.84**	0.08
Item 1	0.03	0.17	0.04	**0.86**
Item 6	0.01	0.12	0.10	**0.91**
Item 11	0.04	0.10	0.13	**0.91**
Eigenvalues	5.23	1.20	1.09	2.38
% of variance	21.83	59.64	76.11	41.59

Boldfaced loadings represent significant loadings on that factor. IEXS, Intuitive Exercise Scale; Factor 1, Emotional Exercise; Factor 2, Body Trust; Factor 3, Exercise Rigidity; Factor 4, Mindful Exercise.

#### 3.2.2 Confirmatory factor analysis

**Figure 1:**
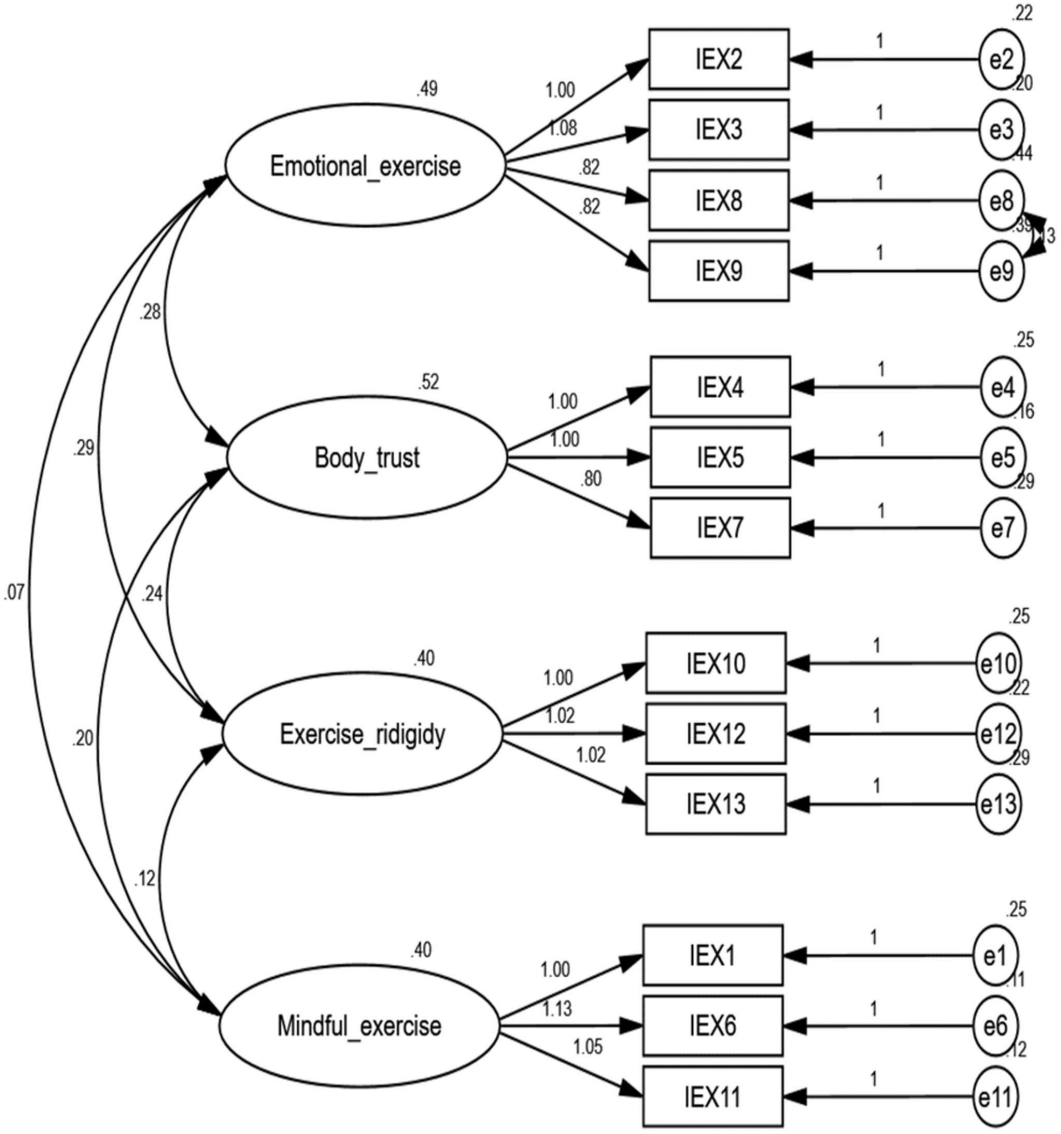
Confirmatory factor analysis of the four-factor model of IEXS-C. Boldfaced loadings indicate significant loadings on the corresponding factor. Parameter estimates next to curved arrows signify factor correlations. Parameter estimates above each straight arrow denote standardized regression weights.

We conducted CFA on the even group (*n* = 315) data. Following established guidelines, we utilized several fit indices to examine the model’s overall fit, including Goodness-of-fit index (GFI), Normed fit index (NFI), Incremental fit index (IFI), Tucker-Lewis index (TLI), Comparative fit index (CFI), and Root mean square error of approximation (RMSEA). Fit values above 0.90 for GFI, NFI, IFI, TLI, and CFI, and an RMSEA value below 0.08, indicate a good fit (Kline, 2023). The CFA mode is depicted in Figure 1. The analysis results showed that χ^2^/df was less than 3, GFI, NFI, IFI, TLI, and CFI were all above 0.9, and RMSEA was below 0.08, confirming that the model met the required fit criteria (DeVellis, 2021). As detailed in Table 3, all standardized factor loadings exceeded 0.5, and the composite reliability (CR) of each factor ranged from 0.831 to 0.896. The average variance extracted (AVE) values for each factor were 0.581, 0.658, 0.621, and 0.742. Following the criteria set by Fornell and Larcker (1981), CR is expected to surpass 0.6 and AVE is supposed to exceed 0.5, which was met for all factors, confirming convergent validity. To assess discriminant validity, we applied the Fornell-Larcker criterion. That is, the square root of the average extracted variance for each dimension was 0.842, 0.788, 0.811, and 0.762, all exceeding the correlation coefficients between dimensions, demonstrating that the measurement model exhibits robust discriminant validity (Kline, 2023). Thus, the IEXS-C shows robust structural validity and satisfies statistical criteria.

**Table 3:** Confirmatory factor analysis of the Chinese version of IEXS (*n* = 315).

Dimensionality	Trails	Subject matter	Standardization factor	S.E.	AVE	CR
Emotional exercise	→	IEX_2	0.829	–	0.581	0.845
		IEX_3	0.864	0.047		
		IEX_8	0.659	0.049		
		IEX_9	0.674	0.047		
Body trust	→	IEX_4	0.824	–	0.658	0.852
		IEX_5	0.874	0.044		
		IEX_7	0.729	0.041		
Exercise rigidity	→	IEX_10	0.787	–	0.621	0.831
		IEX_12	0.807	0.053		
		IEX_13	0.769	0.054		
Mindful exercise	→	IEX_1	0.784	–	0.742	0.896
		IEX_6	0.907	0.046		
		IEX_11	0.888	0.043		
Targets	X^2^/df	RMSEA	GFI	NFI	CFI	TLI
Models	2.87	0.055	0.960	0.964	0.976	0.967
Dimensionality		1	2	3	4	
Mindful exercise (1)		0.842				
Exercise rigidity (2)		0.308	0.788			
Body trust (3)		0.432	0.529	0.811		
Emotional exercise (4)		0.151	0.646	0.549	0.762	

### 3.3 Criterion-related validity analysis

For the purpose of evaluating the criterion-related validity of the IEXS-C, we used the PARS-3, SWLS, PANAS, and SSRS. The outcomes of this analysis can be seen in Table 4.

**Table 4:** Correlations between scales (*n* = 630).

	M ± SD	1	2	3	4	5	6
IEXS-C	47.230 ± 7.481	_					
PARS-3	19.241 ± 20.541	0.255^***^	_				
SWLS	35.753 ± 5.635	0.328^***^	0.187^***^	_			
PA	28.737 ± 7.597	0.287^***^	0.244^***^	0.335^***^	_		
NA	23.252 ± 8.459	0.027	0.099^*^	−0.056	0.534^***^	_	
SSRS	35.753 ± 5.635	0.249^***^	0.151^***^	0.369^***^	0.281^***^	−0.107^**^	_

SD, standard deviation; PARS-3, Physical Activity Rating Scale; SWLS, Satisfaction with Life Scale; PANAS, Positive and Negative Affect Scale; SSRS, Social Support Rating Scale.

^*^p < 0.05, ^**^p < 0.01, ^***^p < 0.001.

### 3.4 Reliability analysis

The revised IEXS demonstrated strong reliability metrics across multiple dimensions. The overall Cronbach’s alpha coefficient for the scale was 0.893, with a Spearman-Brown split-half reliability of 0.827. For the Emotional Exercise dimension, Cronbach’s alpha was 0.852, and the split-half reliability was 0.788. The Body Trust dimension yielded a Cronbach’s alpha of 0.847 and a split-half reliability of 0.880. Similarly, the Exercise Rigidity dimension had a Cronbach’s alpha of 0.860 and a split-half reliability of 0.867. The Mindful Exercise dimension exhibited the highest reliability, with a Cronbach’s alpha of 0.893 and a split-half reliability of 0.903 (see Table 5). Additionally, the intraclass correlation coefficient (ICC) for test–retest scores was 0.854 (*p* < 0.01), while individual items showed test–retest reliability, ranging from 0.346 to 0.706, as detailed in Table 6. Collectively, these findings confirm the scale’s robust reliability across various dimensions and applications.

**Table 5:** Reliability analysis table of the Chinese version of IEXS.

	Emotional exercise	Body trust	Exercise rigidity	Mindful exercise	Total IEXS score
Internal consistency (*n* = 630)	0.852	0.847	0.860	0.893	0.893
Split-half reliability (*n* = 630)	0.788	0.880	0.867	0.903	0.827

**Table 6:** Test–retest results of IEXS-C (*n* = 218).

Item	Pearson correlations
Item_1 当我感觉疼痛时, 我会停下运动。	0.364^***^
Item_2 当我感到有负面情绪(焦虑、悲伤、抑郁)时, 即使我不想锻炼, 我也会锻炼。	0.497^***^
Item_3 当我感到孤独的时候, 即使我不想锻炼, 我也会不自觉地去锻炼。	0.528^***^
Item_4 我相信我的身体会告诉我什么时候锻炼。	0.406^***^
Item_5 我相信我的身体会告诉我该做什么运动。	0.433^***^
Item_6 当我感觉累的时候, 我会停下锻炼。	0.489^***^
Item_7 我相信身体的信号会告诉我该做多少运动。	0.378^***^
Item_8 我通过锻炼来缓解我的负面情绪。	0.706^***^
Item_9 如果我感觉压力很大时, 即使我已经锻炼过了, 我还是会去锻炼。	0.677^***^
Item_10 我将多种体育活动纳入我的锻炼计划中。	0.498^***^
Item_11 当我的身体感到疲劳时, 我就停止锻炼。	0.424^***^
Item_12当我锻炼时, 我喜欢不同类型的体育活动。	0.401^***^
Item_13 我进行多种不同类型的运动。	0.346^***^
Item_14 我通过锻炼来分散自己的注意力, 以避免或摆脱负面情绪。	0.585^***^
Total IEXS	0.854^***^

## 4 Discussion

This research conducts the first psychometric evaluation of the IEXS in the context of Chinese young adults, focusing on its factor structure and applicability. The findings underscore that the revised scale retains its reliability and validity, successfully addressing the unique characteristics of this population. Previous research on the IEXS primarily concentrated on adult populations or people with eating disorders (Reel et al., 2016a; Reel et al., 2016b; Voelker et al., 2021). By extending the scale’s use to a broader demographic, this study bridges an important gap, verifying its utility in assessing intuitive exercise behaviors within a Chinese cultural context. These results not only broaden the scale’s applicability but also reinforce its relevance in diverse socio-cultural settings, emphasizing its potential for future research and practice.

Firstly, this research assessed the content validity of the IEXS, ensuring that the items reflected the cultural nuances of the Chinese measurement environment (DeVellis, 2021). Revising a scale for cross-cultural application requires a careful alignment of linguistic and cultural contexts, particularly taking into account the disparities between Eastern and Western languages and cultures. The back-translation method was employed to maintain conceptual, semantic, and contextual accuracy, reducing potential errors from cultural differences. The final version confirmed semantic consistency between the original text and its translation. Furthermore, the study considered the participants’ understanding of the scale. Misunderstanding items could increase the likelihood of dishonest responses (Nguyen, 2017), thereby compromising the validity of the questionnaire and adversely affecting research outcomes (Zhong et al., 2021). To address this, examples were incorporated into the questionnaire design to enhance comprehension. For instance, question 5, “I trust my body to tell me what kind of exercise to do,” was supplemented with the example: “Today, I feel soreness in my shoulders and neck muscles, so I would do some stretching or relaxation exercises for the shoulders and neck.” These adjustments ensured participants accurately understood and responded to the scale items.

Exploratory and confirmatory factor analyses demonstrated that the IEXS exhibited good construct validity in this study. Two rounds of EFA led to the removal of item 14 from the “Emotional Exercise” factor, resulting in 13 items across four dimensions, consistent with the original scale structure (Reel et al., 2016a; Reel et al., 2016b). The adjustment likely reflects differences in Chinese participants’ exercise habits. While the Emotional Exercise dimension assesses the use of physical activity to regulate negative emotions, many Chinese college students do not actively exercise to prevent or mitigate negative emotions (Liu et al., 2021). Instead, they often engage in physical activity due to external motivations, such as course requirements or credit incentives, rather than intrinsic interest (Pan et al., 2022). This dynamic aligns with the “Crispy College Students” phenomenon, where academic pressures and inadequate exercise facilities discourage regular physical activity (Zhou et al., 2024).

Several factors contribute to this scenario: a lack of internal motivation leading to difficulty sustaining exercise habits (Aphramor, 2010; Ostendorf et al., 2021; Teixeira et al., 2012); external incentives that make physical activity monotonous and rigid (Ostendorf et al., 2021); and short-term approaches that emphasize results over sustainability, often resulting in injuries or adverse physical reactions (Adkins and Keel, 2005; Brown and Summerbell, 2009). Misconceptions about physical exercise, particularly focusing excessively on outcomes, exacerbate the issue (Calogero and Pedrotty-Stump, 2010). This highlights the need for balanced educational strategies emphasizing the role of regular, enjoyable physical activity in maintaining holistic well-being.

In this study, we conducted a CFA on the 4-factor, 13-item IEXS, confirming that all model fit indices met established thresholds, indicating a stable internal structure. The scale’s reliability and factor structure were further evaluated through item analysis. The CR values for each item, along with significant correlations of each item with the overall score, demonstrated strong discriminant validity. To verify the scale’s predictive validity, correlations between IEXS dimensions and the PARS-3 were examined. Our results revealed significant positive correlations, confirming that intuitive exercise dimensions align with physical activity levels. These findings provide robust evidence that the IEXS possesses strong predictive validity.

The IEXS-C demonstrated remarkable internal consistency, achieving a Cronbach’s alpha value of 0.893, exceeding the standard benchmark of 0.80 (Lance et al., 2006). This reliability metric is notably higher than the English version’s Cronbach’s alpha of 0.86 (Reel et al., 2016a; Reel et al., 2016b). Additionally, the test–retest reliability coefficient for the IEXS-C was 0.854, affirming its stability over time. These reliability results support the scale’s utility in educational and intervention contexts. Universities and educational institutions can leverage the IEXS-C as an effective tool for assessing students’ engagement in intuitive exercise, guiding targeted strategies to promote regular physical activity. Such initiatives can enhance students’ overall health and well-being by addressing gaps in exercise habits and providing essential resources and support.

This research has several limitations that warrant attention. First, the sample lacks diversity, as it predominantly consists of college students from universities in the Guangzhou area, limiting the results’ generalizability to broader populations. Future research should incorporate more samples to ensure the scale’s universality and applicability across different demographic groups. Second, cultural differences pose another challenge. As the IEXS-C is a translation of the original English version, cultural nuances might affect respondents’ understanding and interpretation of the scale. These discrepancies could influence its applicability in contexts with distinct cultural backgrounds. Over and above, the study did not evaluate the scale’s practical application in intervention measures, such as its effectiveness in predicting or assessing the influence of exercise interventions on intuitive physical activity. Future studies should pay more attention to validate the scale’s utility in intervention-based settings.

## 5 Conclusion

The findings demonstrate that the IEXS-C serves as a reliable and valid measurement instrument for measuring Chinese college students’ engagement in intuitive physical exercise. This revision represents a significant contribution to advancing China’s efforts to promote sports and healthy exercise practices among college students. The finalized IEXS-C consists of 13 items across four factors: emotional movement, bodily trust, exercise rigidity, and mindful movement. It serves as a valuable tool for assessing participation in intuitive exercise and holds the potential for informing policies and interventions aimed at fostering sustainable and scientifically sound exercise habits in college students.

Analogously, this research establishes that the Chinese version of the IEXS-C demonstrates strong reliability and validity for assessing intuitive exercise behaviors among Chinese college students. By refining the scale to include 13 items across four distinct dimensions—emotional movement, bodily trust, exercise rigidity, and mindful movement—the research highlights its suitability for capturing the nuances of intuitive physical activity in a culturally relevant context. Therefore, this work not only expands the applicability of the IEXS to a new population but also contributes to the promotion of healthy exercise practices and informed intervention strategies within Chinese higher education. The findings underscore the potential of the IEXS-C as a practical tool for fostering a deeper understanding of exercise behaviors and encouraging sustainable, health-focused physical activity among students.

We, therefore, recommend applying the IEXS-C in diverse populations beyond Chinese college students to further validate its generalizability and cultural adaptability. While this study demonstrates the scale’s reliability and validity, its limitations include a geographically restricted sample from the Guangzhou area and the potential for cultural interpretation differences in the translated items. Additionally, the study did not evaluate the scale’s practical application in exercise interventions, leaving its predictive and evaluative utility untested. Future research should address these limitations by including more representative and varied samples, conducting cross-cultural comparisons, and assessing the scale’s effectiveness in predicting or measuring the outcomes of targeted exercise interventions.

## Data Availability

The original contributions presented in the study are included in the article/supplementary material, further inquiries can be directed to the corresponding author.
